# Role of NAD(P)H Oxidase in Superoxide Generation and Endothelial Dysfunction in Goto-Kakizaki (GK) Rats as a Model of Nonobese NIDDM

**DOI:** 10.1371/journal.pone.0011800

**Published:** 2010-07-26

**Authors:** Sachin Gupte, Nazar Labinskyy, Rakhee Gupte, Anna Csiszar, Zoltan Ungvari, John G. Edwards

**Affiliations:** 1 Department of Biochemistry and Molecular Biology, University of South Alabama, Mobile, Alabama, United States of America; 2 Reynolds Oklahoma Center on Aging, University of Oklahoma Health Sciences Center, Oklahoma City, Oklahoma, United States of America; 3 Department of Physiology, New York Medical College, Valhalla, New York, United States of America; Mayo Clinic, United States of America

## Abstract

**Background:**

Cardiovascular disease is the leading cause of mortality in diabetics, and it has a complex etiology that operates on several levels. Endothelial dysfunction and increased generation of reactive oxygen species are believed to be an underlying cause of vascular dysfunction and coronary artery disease in diabetes. This impairment is likely the result of decreased bioavailability of nitric oxide (NO) within the vasculature. However, it is unclear whether hyperglycemia *per se* stimulates NADPH oxidase-derived superoxide generation in vascular tissue.

**Methods and Results:**

This study focused on whether NADPH oxidase-derived superoxide is elevated in vasculature tissue evoking endothelial/smooth muscle dysfunction in the hyperglycemic (169±4 mg%) Goto-Kakizaki (GK) rat. By dihydroethidine fluorescence staining, we determined that aorta superoxide levels were significantly elevated in 9 month-old GK compared with age matched Wistar (GK; 195±6%, Wistar; 100±3.5%). Consistent with these findings, 10^−6^ mol/L acetylcholine-induced relaxation of the carotid artery was significantly reduced in GK rats compared with age matched Wistar (GK; 41±7%, Wistar; 100±5%) and measurements in the aorta showed a similar trend (p = .08). In contrast, relaxation to the NO donor SNAP was unaltered in GK compared to Wistar. Endothelial dysfunction was reversed by lowering of superoxide with apocynin, a specific Nox inhibitor.

**Conclusions:**

The major findings from this study are that chronic hyperglycemia induces significant vascular dysfunction in both the aorta and small arteries. Hyperglycemic induced increases in NAD(P)H oxidase activity that did not come from an increase in the expression of the NAD(P)H oxidase subunits, but more likely as a result of chronic activation via intracellular signaling pathways.

## Introduction 

Of the 24 million diabetic Americans, the vast majority suffer from noninsulin dependent diabetes mellitus or type 2 diabetes, while another 57 million Americans are pre-diabetic. Originally thought to be a metabolic problem, widespread systemic complications are now recognized. Cardiovascular disease is the leading cause of mortality in NIDDM has a complex etiology that operates on several levels to include both atherogenic and myocardial components [1], [2]. The vascular complications include intermittent claudication, atherosclerosis, hypertension, retinopathy, nephropathy, and congestive heart failure. The vascular problems of NIDDM individuals are believed to be traceable to alterations in endothelial function.

Diabetes results in significant impairment of endothelium-dependent vasodilatation in response to acetylcholine or increases in flow. This impairment is likely the result of decreased effectiveness of NO mediated functions within the vasculature. It is unclear if this impairment is the result of decreased NO synthesis or bioavailability as a result of an altered vascular phenotype. NO signaling not only regulates vascular tone, but also inhibits of components of the atherogenic process including platelet aggregation, monocyte adhesion, and vascular smooth muscle migration [3], [4], [5]. Endothelial dysfunction and increased generation of reactive oxygen species are believed to be an underlying cause of vascular dysfunction and coronary artery disease in diabetes. Increased NAD(P)H levels have been observed in the spontaneous diabetic BB rat also a model of type 1 diabetes [6]. We have reported in the Zucker *fa/fa*, a model of type 2 diabetes, an increase in superoxide generation was observed [7]. All of these models have both components of hyperlipidemia, hypercholesterolemia, and hyperglycemia, and it is unclear if hyperglycemia alone may stimulate NADPH oxidase-derived superoxide generation in vascular tissue.

Increased generation of reactive oxygen species is believed to be an underlying cause of vascular dysfunction and coronary artery disease in diabetes. Recent studies have shown that components of NADPH oxidase are up-regulated in type 1 diabetes in the vasculature [8]. However, it is unclear whether hyperglycemia *per se* stimulates NADPH oxidase-derived superoxide generation in vascular tissue. The GK rats are a nonobese model of NIDDM that have elevated fasting glucose, impaired response to glucose, and increased HbA1c levels at an early age [9], [10], [11]. Compared to other diabetic animal models, including the Zucker *fa/fa* and Lepr^db^ mouse, the GK rats are not severely hyperlipidemic or hypercholesterolemic and present as a model of hyperglycemia [7], [12]. Therefore, the objective of this study was to elucidate whether endothelial or smooth muscle dysfunction was evident in the hyperglycemic GK rats and the potential role NADPH oxidase function may have.

## Materials and Methods

### Animal Model

**Figure 1:**
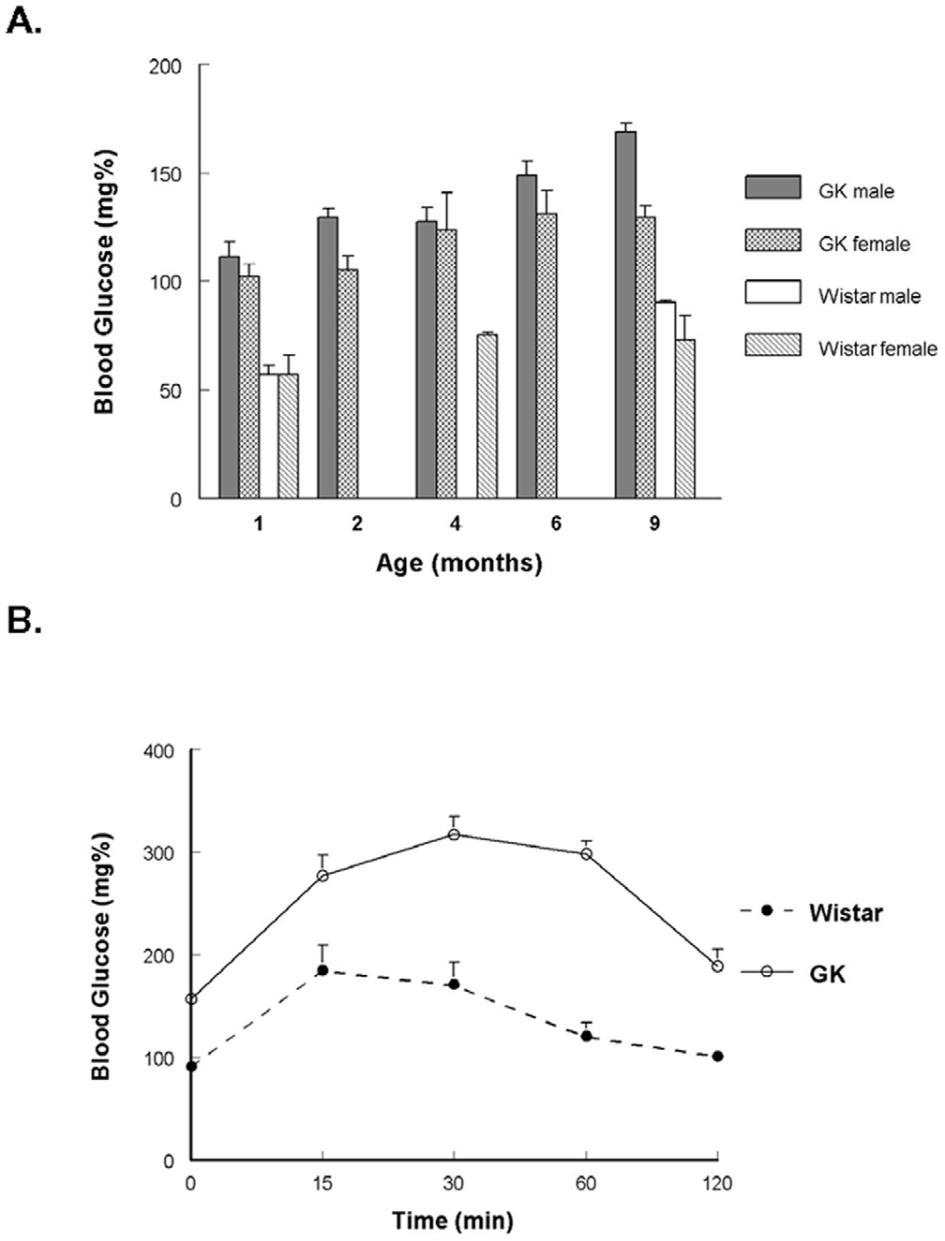
GK rats are hyperglycemic. **A.** Fasting blood glucose from GK and Wistar rats at indicated ages. Samples taken in morning after overnight fast of 16 hours. Values are mean±SEM of 3 to 8 animals. **B.** GK rats have impaired glucose tolerance. Animals were anesthetized with Nembutal (40 mg/kg, *i.p.*). Glucose was injected (1.0 mg/kg *i.p.*) and blood glucose determined at select intervals. Area under the curve (AUC) analysis determined that the GK was significantly (p<.05) increased compared to Wistar following injections (Goto-Kakzaki:12631±1191 n = 8, Wistar: 4902±1247 n = 9 AUC arbitrary units).

Male euglycemic Wistar and diabetic Goto-Kakizaki rats were used throughout this study (with the exception of Figure 1A) [13]. The GK rats are a nonobese model of NIDDM that have elevated fasting glucose, impaired response to glucose, and increased HbA1c levels at an early age [9], [10], [11]. Experimental protocols using animals had approval from the New York Medical College Institutional Animal Care and Use Committee (A3362-01). Animals were maintained in accordance with institutional polices and the Public Health Service (NIH∶PHS) Policy on Humane Care and Use of Laboratory Animals (revised 8/2002).

### Glucose Tolerance Test (GTT)

Glucose was tested following an overnight fast and started between 0930 and 1030 the following morning. The animals were injected with Nembutal (40 mg/kg, *i.p.*), and then at least 15 minutes allotted to achieve a suitable plane of anesthesia. Sterile glucose (1.0 g/kg ***i.p.***) was injected into the abdominal cavity being careful to avoid the g–i tract. Tail vein blood (50 µl) was sampled at selected intervals (pre-injection, 15, 30, 60, 120 min). HbA1c was determined using the A1CNow kits (Bayer Healthcare, Tarrytown NY). Blood glucose was determined using an Accu-Chek monitor (Roche Diagnostics, Indianapolis, IN) calibrated using known standards. At the end of the protocol, the animals were given an addition injection of Nembutal (75 mg/kg, *i.p.*) prior to tissue harvest.

### Vessel isolation and functional studies

The aorta of each animal were carefully exposed and isolated from the surrounding tissues. The vessels were cleaned from the adventitia using an operating microscope and microsurgery instruments. Endothelial function was assessed as previously described [14], [15]. In brief, aortic segments of each animal were cut into ring segments 1.5 mm in length and mounted on 40 µm stainless steel wires in the myographs chambers (Danish Myo Technology A/S, Inc., Denmark) for measurement of isometric tension. The vessels were superfused with Krebs buffer solution (118 mmol/L NaCl, 4.7 mmol/L KCl, 1.5 mmol/L CaCl_2_, 25 mmol/L NaHCO_3_, 1.1 mmol/L MgSO_4_, 1.2 mmol/L KH_2_PO_4_, and 5.6 mmol/L glucose; at 37°C; gassed with 95% air and 5% CO_2_). After an equilibration period of 1 hour during which a optimal passive tension was applied to the rings (as determined from the vascular length-tension relationship), relaxations of pre-contracted (by 10^−6^ mol/L phenylephrine) vessels to acetylcholine (ACh; from 10^−8^ to 10^−6^ mol/L) and the NO donor S-nitrosopenicillamine (SNAP, from 10^−9^ to 10^−5^ mol/L) were obtained.

### Microvascular dilation

NO-mediated microvascular responses were compared by assessing acetylcholine-induced dilation in isolated, pressurized first-order skeletal muscle arterioles, as we previously reported [16].

### Dihydroethidine fluorescence

Production of O_2_^.−^ was determined in segments of the aortas that were used for functional studies. Hydroethidine, an oxidative fluorescent dye, was used to localize superoxide production *in situ* as we previously reported [17]. In brief, vessels were incubated with hydroethidine (3×10^−6^ mol/L; at 37°C for 60 min). The arteries were then washed three times, embedded in OCT medium and cryosectioned. Fluorescent images were captured at ×10 magnification and analyzed using the Zeiss Axiovision imaging software. Ten to fifteen entire fields per vessel were analyzed with one image per field. The mean fluorescence intensities of ethidium–stained nuclei in the endothelium and medial layer were calculated for each vessel. Thereafter, these intensity values for each animal in the group were averaged. Unstained aortas and vessels pre-incubated with PEG-SOD were used for background correction and negative control, respectively.

### Superoxide Activity

In brief, aortas were homogenized in ice-cold buffer (20 mM HEPES-pH 7.4, 0.1 mmol/L EDTA, 1 mM glutathione, 10^−5^ mol/L BH_4_, 1× proteinase inhibitor. Protein concentration was determined by the Bradford method. NADPH oxidase activity, using 5 µM lucigenin, was determined as we have previously described [18]. NOX-dependent activity was determined by the addition of (3×10^−4^ mol/L apocynin, 5×10^−5^ mol/L gp91ss-tat, or 10^−6^ mol/L diphenyleneiodonium (DPI).

### Western Blot Analysis

Tissues were stored at −80°C until used. Aorta samples were homogenized in ice-cold buffer (20 mmol/L HEPES pH7.5, 50 mmol/L NaCl, 1% SDS, 1× protease inhibitor (Sigma-Aldrich, P-8340). Protein concentration was determined by the Bradford method (BioRad reagent). Samples were mixed with Lamelli buffer, heated to 95°C for 5 min, and loaded onto a SDS-PAGE and electrophoresis was performed at room temperature. The gels were blotted onto Hybond-P (Amersham Biosciences, Piscataway NJ) by a semi-dry transfer protocol. Western analysis was performed as described previously [19]. Antibodies used included mouse monoclonal anti-Nox-1, anti-Nox-2, anti-p67phox, and smooth muscle alpha-actin (Transduction Laboratory, San Jose, CA, USA), goat polyclonal anti-Nox-4 and anti-p47phox (Santa Cruz Biotech, Santa Cruz, CA). Antibody dilutions were 1∶500 for all Nox subunits and 1∶2000 for alpha-actin. Antibody binding was visualized using the Amersham ECL *Plus* kit. Band density was quantified using AlphaEaseFC software (AlphaInnotech, San Leando CA).

### RNA Analysis

Total RNA from was isolated using a FASTRNA *ProGreen* Kit (Q-Biogene, Irvine CA). Quantification of mRNA levels was done by QRT-PCR by real-time fluorescent using a Stratagene MX3000p as described previously [19], [20]. The data was normalized by Δ2Ct method using β-actin and the fidelity of the reactions were verified by melting point analysis. Data presented are the mean±SEM with respect to sedentary control values.

### Statistical Analysis

Statistical analyses were performed using NCSS Software (NCSS, Kaysville UT). Where appropriate, student t-test or ANOVA was utilized, post-hoc analysis was done using a Fisher's LSD analysis. Values presented are mean±SEM and statistical significance was set at p<.05.

## Results

At 9 months of age the GK weighed significantly less than the Wistar group (Table 1). When this difference was considered there was no indication of cardiac hypertrophy, but a significant increase in the Kidney/BW ratio was observed. Similar to previous reports the GK rats are both hyperglycemic and have impaired glucose tolerance (Figure 1).

**Table 1:** Morphometric Data.

	Body Wt.	Kidney Wt.	Heart Wt.	Kidney/BW	Heart/BW	HbA1c
	(g)	(g)	(g)			(%)
Wistar	375±19	1.06±0.03	1.14±0.03	2.57±0.05	3.07±0.20	4.90±0.14
GK	232±3	0.80±0.02	0.74±0.01	3.41±0.09	3.17±0.04	7.33±0.48
	p<.05	p<.05	p<.05	p<.05	ns	p<.05

**Figure 2:**
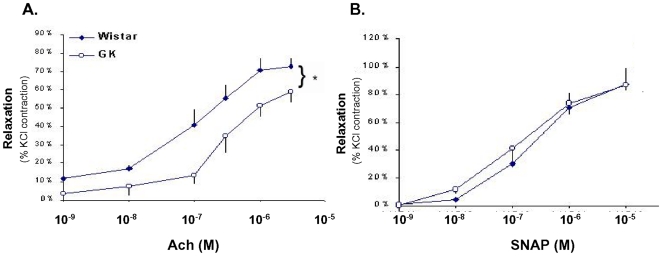
Vasorelaxation of small arteries (<150 µm) from 9 month-old GK or Wistar rats is endothelia dependent. **A.** Diabetes significantly decreased acetylcholine-induced dilation (≥10^−6^ mol/L) in GK compared to Wistar. **B.** Diabetes did not alter the response to SNAP (a nitric oxide donor). Values are mean±SEM of 4 animals. * p<.05 compared to Wistar.

**Figure 3:**
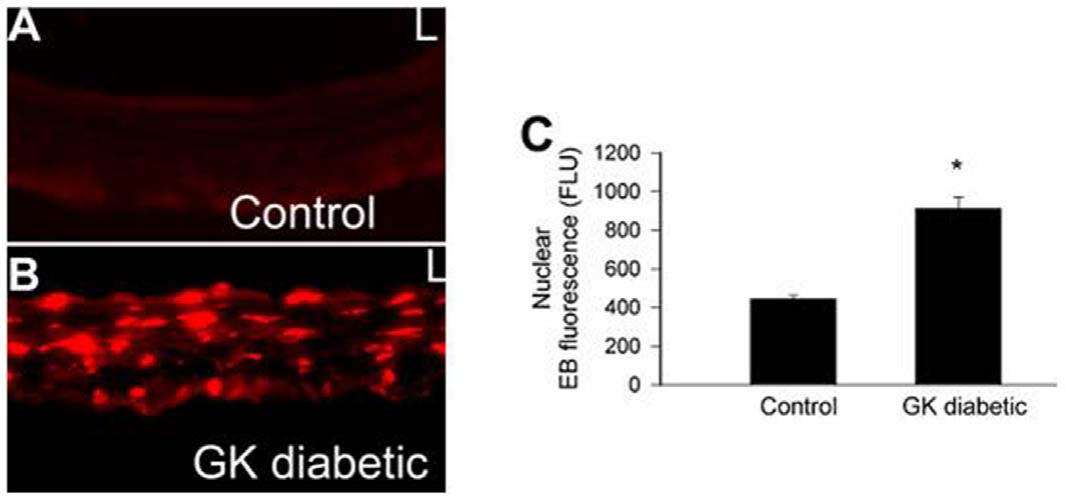
DHE fluorescence staining for superoxide. **A.** 9 month-old Wistar aorta, **B.** 9 month-old GK aorta. **C.** Quantification of DHE staining. Images were collected with a CoolSnap CCD camera attached to an Olympus BX60 microscope. Values are mean±SEM of 4 animals. * p<.05 compared to Wistar.

An impaired vasodilator response has been observed in several models of diabetes. In the present study we observed that acetylcholine induced vasorelaxation was impaired in the small arteries of the diabetic GK (Figure 2A), while, no differences between control Wistar and diabetic GK were observed in response to SNAP(a NO donor) (Figure 2B). These results suggest that diabetes impaired NO bioavailability. One underlying mechanism for impaired NO bioavailability is superoxide reaction to convert NO to ONOO and H_2_O. TO determine if superoxide was increased in the diabetic artery, tissue was stained using DHE. As shown in Figure 3, we observed significant increases in the present of superoxide.

**Figure 4:**
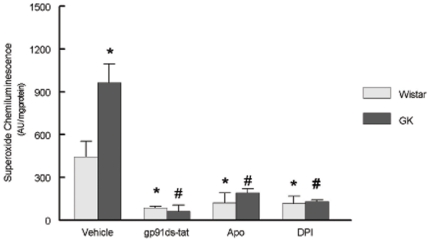
Diabetes significantly increased superoxide production in aortic tissue. A small portion of the abdominal aorta was resected from nine month old GK and Wistar rats. Superoxide was determined by lucigenin chemiluminescence, using 5×10^−6^ mol/L lucigenin and 2×10^−4^ mol/L NADPH. To determine if superoxide generation was derived specifically from NADPH tissue samples were incubated with three different inhibitors of NADPH; apocynin (APO), diphenyleneiodoium (DPI), and gp91ds-tat. Values are mean±SEM of 5 animals. * p<.05 compared to Wistar control, #p<.05 compared to GK control.

Similar to our earlier findings in the myocardium [7], NADPH directed lucigenin chemiluminescence was significantly increased in the diabetic aorta compared to the Wistar controls (Figure 4). No differences in NADH directed lucigenin chemiluminescence were observed (data not shown). The presence NAD(P)H inhibitors including pg91^ds-tat^, apocynin, and diphenyleneiodonium all reduced chemiluminescence and no differences between the Wistar and GK groups were observed (Figure 4).

**Figure 5:**
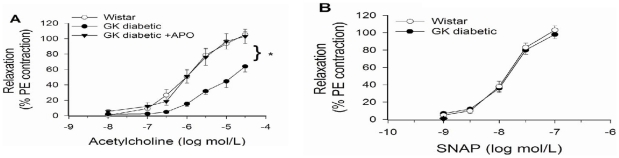
Apocynin restores vasorelaxation of the diabetic aorta. **A.** Diabetes significantly decreased acetylcholine-induced dilation ≥1 µM in GK compared to Wistar. Apocynin (APO) was used to inhibit NADPH-dependent generation of superoxide. **B.** Diabetes did not alter the response to SNAP (a nitric oxide donor). Values are mean±SEM of 5 animals. * p<.05 compared to Wistar.

Superoxide may be derived from several sources including mitochondria, monomeric eNOS, and NAD(P)H oxidase. We have previously observed that apocynin (an NAD(P)H oxidase inhibitor) decrease NAD(P)H-directed superoxide generation in the myocardium [21]. To determine the potential role of NAD(P)H derived superoxide on vascular function, the aorta was preincubated in apocynin. In response to acetylcholine induced vasorelaxation, no differences in vasorelaxation between diabetic and control aortas were observed (Figure 5). This would suggest that NAD(P)H derived superoxide generation may have been the underlying cause of vasoactive dysfunction in GK arteries.

**Figure 6:**
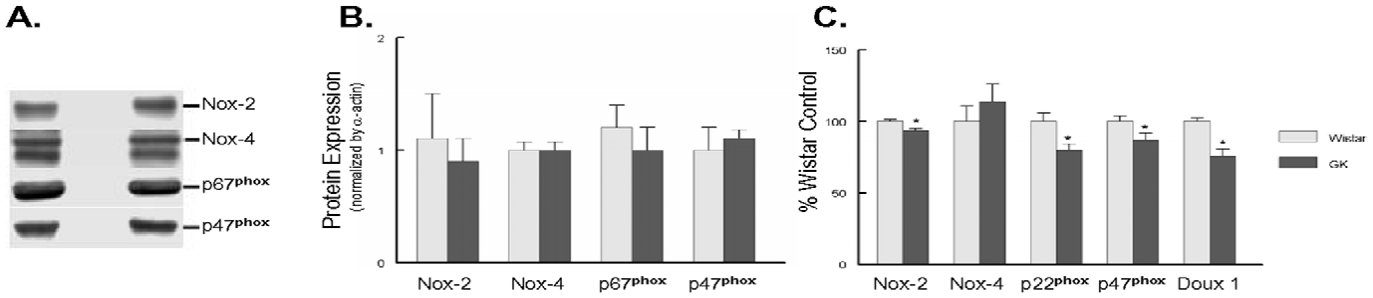
Diabetes did not significantly alter NOX protein expression. **A.** Representative western blot, **B.** Quantification of western blot data normalized to alpha-actin. No differences were observed between the GK and Wistar groups. **C.** QRT-PCR of aortic-mRNA. Values are mean±SEM of 4–7 animals. * p<.05 compared to Wistar.

NAD(P)H oxidase consisting of 6 subunits and regulation of its activity is a complex event that we only now just beginning to understand. One possibility is an increase in the presence of the responsible components. Etoh et al. reported that the expression of both Nox4 and p22phox were increased in a STZ-induced model of diabetes [8]. To that end we have examined expression of NAD(P)H oxidase components at both the protein and mRNA levels. We found no significant differences in expression between the Wistar and GK aortas (Figure 6).

## Discussion

The GK rats are a nonobese model of NIDDM that have elevated fasting glucose, impaired response to glucose, and increased HbA1c levels at an early age [9], [10], [11]. Compared to other animal models of diabetes, the GK rats are not hyperlipidemic or hypercholesterolemic and present as a model of hyperglycemia. We have previously determined that GK hyperglycemia is progressive with age and that exercise training will significantly lower fasting blood glucose (sedentary; 205±9, exercise trained; 174±9 mg%) [20]. Although not hypertensive, the GK rats exhibit a small but significant increase MAP and systolic blood pressure compared to their Wistar controls [22]. GK rats present with many diabetic related complications observed in human diabetic patients, including reduced nerve conduction velocity and progressive renal involvement with thickening of the glomerular basement membranes [10], [13], [23], [24], [25], [26], [27]. It has been suggested that impaired pancreatic mitochondrial function may partially explain the depressed insulin release [28], [29]. Whether this is related to a shift in mitochondrial antioxidative capacity resulting in accelerated apoptosis is unclear [30]. Beta-cell mass and beta-cell replication is decreased in the GK pancreas and as such the GK rat may represent a model of beta-cell degradation for human NIDDM [31], [32], [33]. Genetic linkage analysis of the Goto-Kakizaki rats has localized different quantitative trait loci to those involved in diabetes [33], [34], [35], [36], [37]. Some regions show remarkable synteny homology to the diabetic loci found within human chromosome 1q21–25 [34], [35]. More recently Rosengren et. al. identified a polymorphism in the GK that resulted overexpression of the α2A-adrenergic receptor leading to depressed insulin sensitivity [37]. This polymorphism was also associated with a decreased insulin sensitivity in humans supporting the concept decreased insulin sensitivity may be one contributing cause of NIDDM, further linking the GK model to the human condition.

Diabetes impacts the vasculature on several levels. Increased generation of reactive oxygen species leading to endothelial dysfunction is believed to be the major cause of vascular dysfunction and coronary artery disease in diabetes. The GK rats represent a model to examine the role of hyperglycemic in diabetes. The major findings from this study are that chronic hyperglycemia induces significant vascular dysfunction in both the aorta and small arteries. Our studies indicate that NAD(P)H oxidase accounts for a significant portion of intracellular ROS in the vasculature. Hyperglycemic induced increases in NAD(P)H oxidase activity did not come from an increase in the expression of the NAD(P)H oxidase subunits, but more likely as a result of chronic activation via intracellular signaling pathways.

Endothelium-dependent macrovascular dysfunction has been observed in several models of diabetes and our results are similar to others for the larger conduit vessels [22], [38], [39]. Decreased endothelial-dependent relaxation of GK aorta and small skeletal muscle arteries (Figs. 2 & 5), suggest that endothelium-derived NO is inactivated presumably by hyperglycemia-induced oxidative stress. Although not hypertensive, the GK rats have a small but significant increase in MAP and systolic blood pressure compared to their Wistar controls, and have found to be salt sensitive [22]. Our findings are consistent with an increase in blood pressure as a function of vascular pathology.

NAD(P)H oxidase, the mitochondrial electron transport chain, and dysfunctional nitric oxide synthase are the three major sources of ROS within the cell. Although these sources are thought of as separate entities, recent papers have suggested significant interactions [40], [41], [42], [43], [44]. Any source of superoxide will exacerbate the conversion of NO to ONOO^−^ and uncoupled eNOS and increased iNOS expression have both been shown to contribute to oxidant stress in diabetes [40], [41], [45]. In obese models of NIDDM, NAD(P)H oxidase derived ROS is increased, and it has been suggested that elevated vasculature ROS contributes to mitochondrial dysfunction in the GK rat [7], [46], [47]. NOX4 is constitutively active not requiring activation by p47^phox^ or p67^phox^ and its expression is increased in diabetes and aging [42], [44]. Subcellular localization of NOX4 is cell type dependent with NOX4 being localized to the mitochondria in cardiomyocytes, in the nucleus of endothelial cells, and at focal adhesions within vascular smooth muscle [42], [43], [44], [48], [49]. Within vascular smooth muscle, NOX4 is involved in maintenance of the smooth muscle phenotype and localization to focal adhesions suggests its participation in cell migration [49], [50]. Localization of NOX4 to the endothelial nuclei could significantly accelerate oxidant stress induce DNA modifications [49]. In the present study, we find, using an animal model of chronic hyperglycemia, significant increases ROS generation via elevated NAD(P)H oxidase activity. The NAP(P)H inhibitors apocynin as well as gp91^ds-tat^, and DPI all reduced superoxide generation by NAD(P)H oxidase in aortic extracts. Blockage of NAD(P)H oxidase by apocynin restored endothelial dependent vasorelaxation. Although our present results clearly demonstrated NADPH oxidase impacts on endothelial dependent vasculature function, future work to more clearly examine diabetic induced changes in NADPH oxidase by cell type and subcellular local are warranted.

Chronic hyperglycemia did not increased expression of several components of the NAD(P)H oxidase system and this was observed at both the protein and mRNA levels These results are similar to the Zucker fa/fa/rat (type 2) but differ from that observed by Bitnar et.al who observed increases in p47^phox^ in younger GK rats [8], [51], [52]. Other studies examining T1D models observed significant increases in NAD(P)H oxidase subunit expression in the heart and the vasculature suggesting that the pathogenic mechanisms are not necessarily similar in all models of diabetes [41], [53] In the vasculature, the increase was evident after 4 weeks but not 16 weeks of diabetes suggesting that altered NAD(P)H oxidase expression may be a transitional event [53]. The increased NAD(P)H oxidase activity observed in the present study may have arisen as a function of chronic activation of the oxidase, either by increased substrate presentation or increased turnover. Kitahara et.al reported that G6PD activity is significantly elevated in GK rat liver and we have observed similar increases in the myocardium from the Zucker fa/fa rat [51], [54]. The primary function of G6PD is to produce NADPH within the cells and its chronic activation will elevate NAD(P)H levels. In the Zucker fa/fa rat, chronic activation of glucose-6-phosphate dehydrogenase (G6PD) was brought about via a Src kinase [51]. More recently, Liu reported that high glucose will increased p47phox phosphorylation leading to activation of NAD(P)H oxidase [55]. Others have reported that high glucose will enhance p47phox translocation into the membrane promoting activation of NAD(P)H oxidase [56]. Collectively these reports suggest that NAD(P)H oxidase activity may be the result of increased activation.

The GK rats are a model of type II diabetes that presents with hyperglycemia but without severe hyperlipidemia. We have found that endothelial-dependent dysfunction was observed in both large and small arteries. Endothelial dysfunction was brought about as a result of increased intracellular superoxide generation that was likely to be the result of chronic activation of NAD(P)H oxidase activity.
